# Kidney transplantation in children and adolescents with C3 glomerulopathy or immune complex membranoproliferative glomerulonephritis: a real-world study within the CERTAIN research network

**DOI:** 10.1007/s00467-024-06476-5

**Published:** 2024-08-07

**Authors:** Christian Patry, Nicholas J. A. Webb, Manuel Feißt, Kai Krupka, Jan Becker, Martin Bald, Benedetta Antoniello, Ilmay Bilge, Bora Gulhan, Julien Hogan, Nele Kanzelmeyer, Ozan Ozkaya, Anja Büscher, Anne-Laure Sellier-Leclerc, Mohan Shenoy, Lutz T. Weber, Alexander Fichtner, Britta Höcker, Matthias Meier, Burkhard Tönshoff

**Affiliations:** 1 Heidelberg University, Medical Faculty, Department of Pediatrics I, University Children‘s Hospital Heidelberg, Im Neuenheimer Feld 430, Heidelberg, 69120 Germany; 2 Novartis Pharma AG, Basel, Switzerland; 3 Institute of Medical Biometry, University of Heidelberg, Heidelberg, Germany; 4 Institute of Pathology, University Hospital of Cologne, Cologne, Germany; 5 Klinikum Stuttgart, Olgahospital, Stuttgart, Germany; 6 Laboratory of Immunopathology and Molecular Biology of the Kidney, Pediatric Research Institute, Department of Women’s and Children’s Health, Padua University Hospital, Padua, Italy; 7 Koç University Hospital, Koç University School of Medicine, Istanbul, Turkey; 8 Department of Pediatric Nephrology, Hacettepe University, Faculty of Medicine, Ankara, Turkey; 9 Robert Debre Hospital, Department of Pediatric Nephrology, Dialysis, Transplantation, Paris, France; 10 Medical School of Hannover, Clinic of Pediatric Nephrology, Hepatology and Metabolic Diseases, Hannover, Germany; 11 İstinye University Hospital, İstinye University School of Medicine, Istanbul, Turkey; 12 University Hospital of Essen, Department of Pediatrics II, University of Duisburg-Essen, Essen, Germany; 13 Service de Néphrologie Rhumatologie Dermatologie Pédiatrique, Centre de Référence Maladies Rénales Rares “Néphrogones”, Hospices Civils de Lyon, Lyon, France; 14 Royal Manchester Children’s Hospital, Manchester, United Kingdom; 15 Children’s and Adolescents’s Hospital, University Hospital of Cologne, Faculty of Medicine, University of Cologne, Cologne, Germany

**Keywords:** Complement 3 glomerulopathy, Immune complex membranoproliferative glomerulonephritis, Recurrence, Pediatric kidney transplantation, Graft survival

## Abstract

**Background:**

Complement 3 glomerulopathy (C3G) and immune complex membranoproliferative glomerulonephritis (IC-MPGN) are ultra-rare chronic kidney diseases with an overall poor prognosis, with approximately 40–50% of patients progressing to kidney failure within 10 years of diagnosis. C3G is characterized by a high rate of disease recurrence in the transplanted kidney. However, there is a lack of published data on clinical outcomes in the pediatric population following transplantation.

**Methods:**

In this multicenter longitudinal cohort study of the Cooperative European Paediatric Renal Transplant Initiative (CERTAIN) registry, we compared the post-transplant outcomes of pediatric patients with C3G (*n* = 17) or IC-MPGN (*n* = 3) with a matched case–control group (*n* = 20).

**Results:**

Eleven of 20 children (55%) with C3G or IC-MPGN experienced a recurrence within 5 years post-transplant. Patients with C3G or IC-MPGN had a 5-year graft survival of 61.4%, which was significantly (*P* = 0.029) lower than the 5-year graft survival of 90% in controls; five patients with C3G or IC-MPGN lost their graft due to recurrence during this observation period. Both the 1-year (20%) and the 5-year (42%) rates of biopsy-proven acute rejection episodes were comparable between patients and controls. Complement-targeted therapy with eculizumab, either as prophylaxis or treatment, did not appear to be effective.

**Conclusions:**

These data in pediatric patients with C3G or IC-MPGN show a high risk of post-transplant disease recurrence (55%) and a significantly lower 5-year graft survival compared to matched controls with other primary kidney diseases. These data underscore the need for post-transplant patients for effective and specific therapies that target the underlying disease mechanism.

**Graphical abstract:**

**Figure:**
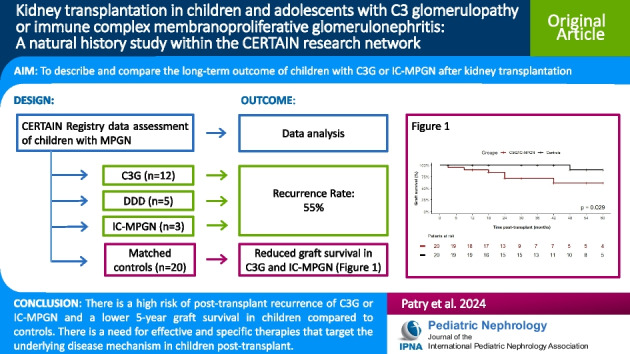
A higher resolution version of the Graphical abstract is available as Supplementary information

**Supplementary Information:**

The online version contains supplementary material available at 10.1007/s00467-024-06476-5.

## Introduction

Complement 3 glomerulopathy (C3G), an ultra-rare chronic kidney disease, is associated with dysregulation of the complement alternative pathway (AP) in plasma and the glomerular microenvironment, thereby resulting in accumulation of C3 and its split products in the glomerulus [1, 2]. C3G is caused by acquired and/or genetic abnormalities affecting the complement pathway, such as nephritic factors, or genetic variants in key AP complement genes. The overall prognosis is poor with approximately 40–50% of patients progressing to kidney failure within 10 years of diagnosis. Studies have reported that 40–50% of C3G patients are diagnosed under 18 years of age, and patients are generally under the age of 40 [3–8]. There is currently no approved treatment for C3G, in particular, none specifically targeting the underlying complement-mediated pathophysiology; pharmacological interventions have shown inconsistent efficacy accompanied by safety concerns.

One of the major challenges in the treatment of patients with C3G is the high rate of disease recurrence in the transplanted kidney, which frequently leads to graft loss and the requirement for maintenance dialysis and/or re-transplantation [9]. There is, however, both a lack of published data on clinical outcomes in this population following successful kidney transplantation (KTx), particularly in the pediatric population, as well as considerable variation in the reported outcomes.

Immune complex membranoproliferative glomerulonephritis (IC-MPGN) is an ultra-rare, fast-progressing kidney disease, which can be idiopathic (primary IC-MPGN) or may be due to secondary causes such as chronic infections and autoimmune diseases [10]. Although IC-MPGN is a separate disease as per the current classification, it has key similarities to C3G, which is also typically characterized by membranoproliferative or mesangioproliferative histopathological patterns. Immunofluorescence (IF) staining in IC-MPGN shows co-dominant immunoglobulin staining with C3, while C3G has predominant complement-3 staining [11, 12]. Similar to C3G, dysregulation of the AP of the complement system is also strongly implicated in the disease pathogenesis of IC-MPGN [13] with similar incidences of both complement autoantibodies and gene mutations in the two conditions. There are limited epidemiological data on IC-MPGN, with no good incidence and prevalence data available; however, several studies indicate approximately equal numbers of primary IC-MPGN and C3G patients [7, 13, 14] pointing towards an incidence of 1–2 per million per year [4]. Like C3G, IC-MPGN is frequently diagnosed in childhood and adolescence, with a median age at diagnosis of around 21 years [15, 16] and is characterized by a high risk of progression to kidney failure [13, 15]. In IC-MPGN patients who undergo KTx, the disease can also recur. Although some evidence suggests post-transplant recurrence may be less common in IC-MPGN than in C3G, outcomes are nevertheless poor in both [7]. Patients who have disease recurrence are at high risk of subsequent kidney graft failure.

There is a significant paucity of clinical outcome data in patients with C3G and IC-MPGN following KTx. This is particularly relevant for children and adolescents, who make up around one half of the prevalent C3G population. The available published patient series are all small, and there is great variation in the reported rates of disease recurrence and impact of this on hard clinical outcomes, including graft loss. The primary objective of this observational registry study was therefore to describe the outcome of children and adolescents with C3G or IC-MPGN following KTx and to compare their outcome with a matched control group of children with other primary kidney diseases that are not associated with the development of recurrent disease following transplantation.

## Materials and methods

### Patients and follow-up

This retrospective, multicenter, longitudinal cohort study included KTx recipients in pediatric care (younger than 21 years at transplantation, according to the North American Pediatric Renal Trials and Collaborative Studies (NAPRTCS) patient recording practice [17]) at the time of KTx enrolled in the Cooperative European Paediatric Renal Transplant Initiative (CERTAIN) registry. The CERTAIN registry collects detailed longitudinal clinical and laboratory data and applies rigorous validity-checking procedures (www.certain-registry.eu). A comprehensive description of the CERTAIN registry including the data validation process has been published previously [18, 19]. Due to its detailed, comprehensive data capture, this registry allows an in-depth characterization of specific patient cohorts.

Participation in the CERTAIN registry is approved by the ethics committee at each center. Informed consent must be obtained from the parents or legal guardians prior to enrollment, with assent from patients when appropriate for their age. The time points of data collection and the corresponding time intervals were as follows: baseline (pretransplant), at months 1, 3, 6, 9, and 12, and every 6 months thereafter. All procedures and immunosuppressive regimens were performed according to local institutional protocols. Anthropometric, clinical, and biochemical data were collected as part of a routine follow-up at each center.

Data collection from CERTAIN for this study was based on the following inclusion criteria: patient age < 21 years at the time of transplantation and a documented primary kidney disease of either C3G (subdivided into C3 glomerulonephritis (C3GN) and Dense Deposit Disease (DDD)) or IC-MPGN. Exclusion criterion was graft loss immediately after KTx due to reasons other than recurrent primary kidney disease. Patients received their KTx between 2006 and 2022. Specific data collection for this analysis was performed according to a defined protocol (see Supporting Information in the online version of this article). The study was performed in accordance with the Declaration of Helsinki and the Declaration of Istanbul on Organ Trafficking and Transplant Tourism. The study was designed, analyzed, and reported according to the STROBE guidelines (https://www.strobe-statement.org).

### Laboratory measurements and definitions

All laboratory values were measured locally and data were reported to the CERTAIN registry. eGFR was calculated according to the revised Schwartz formula: eGFR = (ml/min per 1.73 m^2^) = 0.413 × [height (cm)/serum creatinine (mg/dl)] [20]. Patients who had been classified according to the old MPGN types 1, 2, and 3 system were reclassified as C3G (C3GN or DDD) or IC-MPGN. The diagnosis of recurrence of C3G or IC-MPGN post-transplant was established by graft biopsy. All histopathology reports of the native kidney biopsies and the kidney allograft in case of suspected recurrence were re-evaluated by an experienced nephropathologist (J. B.) and reassessed according to a classification scheme [12] to ensure consistent evaluation of histopathology data. We identified matched control patients with primary kidney diseases other than C3GN, DDD, or IC-MPGN. Each of the matched control patient’s primary kidney disease was non-glomerular. Exclusion criteria were primary kidney diseases with a risk of recurrence post-transplant such as atypical HUS, lupus nephritis, or IgA nephropathy. The matching criteria were as follows: (i) recipient age at KTx stratified into the three age groups 0–5.9, 6–11.9, and 12–21 years, (ii) comparable immunosuppressive regimen for induction therapy and at day 30 post-transplant, (iii) number of HLA mismatches, (iv) donor type (living or deceased donor). These patients had received their kidney allograft in the same era between the years 2006 and 2023. In case of multiple matching partners per case, one was chosen blinded and at random.

### Statistical analysis

The program R (version 4.2.0) was used for data analysis. Patient and transplant characteristics: categorical parameters are presented as the number and percentage of patients, while results for continuous variables are expressed as mean ± SD or as median and first and third quartile, as appropriate. Event rates of time-to-event endpoints were calculated using the Kaplan–Meier estimator. Continuous variables were compared using either Welch’s *t*-test or Mann–Whitney *U*-test, as appropriate. Categorical variables were compared using chi-squared tests. For time-to-event variables, the log-rank test was used. Since this is an exploratory analysis, we did not adjust for multiplicity and *p*-values have to be interpreted in a descriptive sense. A *p* value < 0.05 was considered statistically significant.

## Results

For this specific analysis, 20 patients with C3G (*n* = 17) or IC-MPGN (*n* = 3) who had undergone KTx were documented in the CERTAIN registry. Baseline characteristics of these patients are shown in Table 1. The median age at diagnosis of the native kidney disease (native biopsy diagnosis) was 9.4 years for C3G (IQR, 6.8–13.1 years) and 8.2 years for IC-MGPN (IQR, 4.5–8.8 years). Fifteen patients (C3GN, *n* = 8; DDD, *n* = 4; IC-MPGN, *n* = 3) had received some form of immunosuppressive therapy prior to reaching kidney failure. Six of 20 (30%) patients received a KTx from a living-related donor, and 14 of 20 patients (70%) from a deceased donor. Ten of 20 patients (50%) received induction therapy, either with the interleukin 2 receptor antagonist basiliximab (*n* = 6), or anti-thymocyte globulin (*n* = 4). For immunosuppressive maintenance therapy, the majority of patients received a combination of the calcineurin inhibitor tacrolimus in conjunction with mycophenolate (mycophenolate mofetil (MMF) or mycophenolate sodium (MPS)) (Table 2).

**Table 1:** Baseline characteristics of patients with C3G or IC-MPGN prior to kidney transplantation

ID	Disease subtype	Sex	Age at onset (years)	Autoantibodies against complement factors	Complement mutations or genetic variants	Immunosuppressive therapy	Complement-targeted therapy	Plasma exchange or immunoadsorption
1	C3GN	Female	13.1	ND	C3	Steroids, CNI, MMF	Eculizumab	No
2	C3GN	Male	4.8	ND	C3	None	Eculizumab	No
3	C3GN	Male	9.7	C3Nef	C3	Steroids, MMF, rituximab	Eculizumab	Yes
4	C3GN	Male	14.3	ND	ND	ND	ND	NR
5	C3GN	Male	15.0	ND	ND	None	Eculizumab	NR
6	C3GN	Male	9.3	ND	CFI	None	No	No
7	C3GN	Female	9.2	ND	ND	Steroids, MMF, rituximab	No	No
8	C3GN	Female	13.4	No autoantibodies found	ND	None	No	No
9	C3GN	Female	16.3	C3Nef	ND	None	No	NR
10	C3GN	Female	3.9	C3Nef	ND	Steroids, CYC	Eculizumab	No
11	C3GN	Female	6.5	C3Nef	ND	Steroids, CYC, MMF, rituximab	Eculizumab	Yes
12	C3GN	Male	10.4	No autoantibodies found	ND	Steroids, CNI, CYC, MMF	Eculizumab	Yes
13	DDD	Female	9.4	C3Nef	CFH	Steroids, CYC, rituximab	No	Yes
14	DDD	Male	3.8	ND	C3	Steroids, CNI	No	No
15	DDD	Female	6.8	C3Nef, anti-CFB, anti-C3b	ND	None	No	No
16	DDD	Female	9.6	C3Nef, anti-CFH	ND	Steroids, CYC, rituximab	No	Yes
17	DDD	Female	8.8	C3Nef	ND	Steroids	No	No
18	IC-MPGN	Male	8.2	C3Nef	ND	Steroids	No	No
19	IC-MPGN	Male	11.0	No autoantibodies found	ND	Steroids	No	No
20	IC-MPGN	Female	9.4	ND	ND	Steroids, CNI, MMF	No	NR

*C3G*, C3 glomerulopathy; *C3GN*, C3 glomerulonephritis; *C3Nef*, C3 nephritic factor; *CFH*, complement factor H; *CFB*, complement factor B; *CFI*, complement factor I; *CNI*, calcineurin inhibitor; *CYC*, cyclophosphamide; *IA*, immunoadsorption; *KTx*, kidney transplantation; *MMF*, mycophenolate mofetil; *ND*, not determined; *NR*, not reported

**Table 2:** Comparison of baseline characteristics of patients with C3G or IC-MPGN and matched controls

	C3G/IC-MPGN cohort, *N* = 20	Matched controls, *N* = 20	*P* value
Age at KTx (year), mean ± SD	13.8 ± 3.9	12.5 ± 4.0	0.315^a^
Male sex, *n* (%)	9 (45.0)	12 (60.0)	0.342^b^
Caucasian, *n* (%)	19 (95.0)	20 (100.0)	0.311^b^
Disease subtypes of the C3G/IC-MPGN cohort, *n* (%)			
C3 glomerulonephritis	12 (60.0)	NA	NA
Dense deposit disease	5 (25.0)	NA	
Primary IC-MPGN	3 (15.0)	NA	
Primary kidney diseases in controls, *n* (%)			
Kidney dysplasia/hypoplasia	NA	13 (65.0)	NA
Nephronophthisis	NA	5 (25.0)	
Renal vein thrombosis	NA	1 (5.0)	
Diarrhea-associated HUS	NA	1 (5.0)	
Previous KTx, *n* (%)			
0	15 (75.0)	17 (85.0)	0.300^b^
1	5 (25.0)	2 (10.0)	
2	0 (0.0)	1 (5.0)	
HLA-A mismatches			
0	5 (25.0)	5 (25.0)	> 0.999^b^
1	10 (50.0)	10 (50.0)	
2	5 (25.0)	5 (25.0)	
HLA-B mismatches			
0	3 (15.0)	3 (15.0)	> 0.999^b^
1	15 (75.0)	15 (75.0)	
2	2 (10.0)	2 (10.0)	
HLA-DR mismatches			
0	4 (20.0)	4 (20.0)	> 0.999^b^
1	14 (70.0)	14 (70.0)	
2	2 (10.0)	2 (10.0)	
Donor-related data			
Donor age (years), mean ± SD	27.4 ± 19.8	28.5 ± 18.2	0.852^a^
Male sex, *n* (%)	14 (70.0)	11 (55.0)	0.327^b^
Living-related donor, *n* (%)	6 (30.0)	6 (30.0)	> 0.999^b^
Transplantation-related data			
Cold ischemia time (min), mean ± SD	646 ± 443	598 ± 460	0.739^a^
Warm ischemia time (min), mean ± SD	39.3 ± 14.3	66.0 ± 83.3	0.344^a^
Induction therapy			
Anti-thymocyte globulin, *n* (%)	4 (20.0)	1 (5.0)	0.151^b^
IL-2-receptor antagonists, *n* (%)	6 (30.0)	7 (35.0)	0.736^b^
Maintenance therapy at day 30 post-transplant			
Calcineurin inhibitor, *n* (%)	20 (100.0)	20 (100.0)	> 0.999^b^
Mycophenolic acid, *n* (%)	17 (85.0)	17 (85.0)	> 0.999^b^
mTOR inhibitor, *n* (%)	2 (10.0)	2 (10.0)	> 0.999^b^
Glucocorticoids, *n* (%)	18 (90.0)	18 (90.0)	> 0.999^b^

*IL-2*, interleukin 2; *KTx*, kidney transplantation; *NA*, not applicable

Statistical tests: ^a^parametric *t*-test; ^b^chi-square test

Sixteen patients were screened for complement-associated mutations and/or autoantibodies. Of these 16 patients, 9 tested positive for the presence of C3 nephritic factor and one had an autoantibody to factor H. Further, 6 (C3GN, *n* = 4; DDD, *n* = 2) of these 16 patients had an identified genetic abnormality in the complement AP. Four patients had variants in the gene encoding complement factor 3, one patient had a mutation in the gene encoding complement factor I, and one patient had a mutation in the gene encoding complement factor H.

Data from these patients with C3G or IC-MPGN were compared to a matched case-controlled group (*n* = 20) derived from the CERTAIN registry. Demographic data and baseline characteristics such as recipient age, sex, ethnicity, number of previous transplantations, number of HLA-A, HLA-B, and HLA-DR mismatches, donor age, donor sex, rate of KTx from living-related donors or deceased donors, duration of the respective cold and warm ischemia time, and immunosuppressive induction and maintenance therapy at day 30 post-transplant were comparable between the two cohorts (Table 2).

**Fig. 1:**
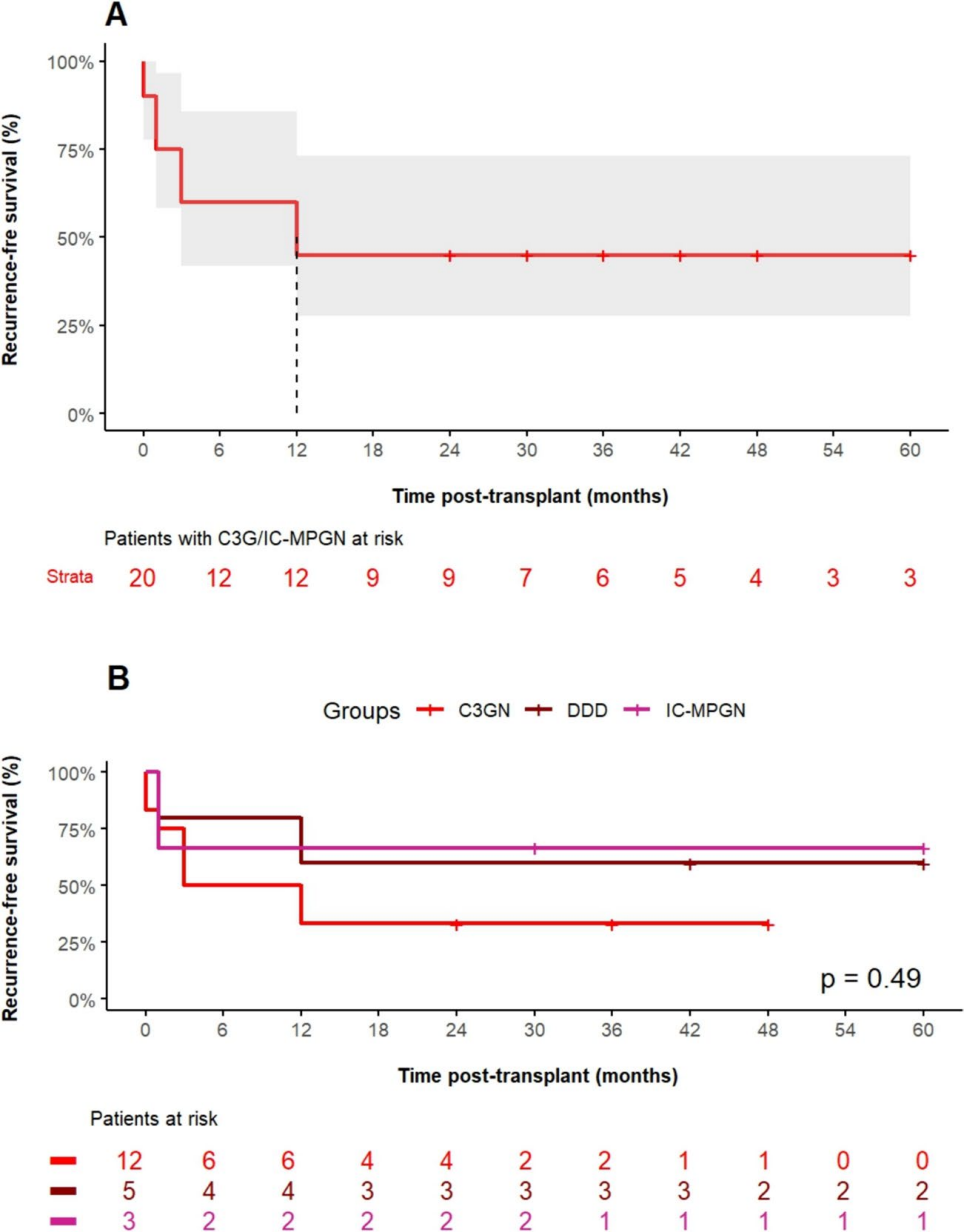
**A** Overall recurrence-free kidney allograft survival over 60 months post-transplant in patients with C3 glomerulopathy (*n* = 17) or immune complex membranoproliferative glomerulonephritis (*n* = 3). The median time of survival without recurrence is marked by a vertical line. **B** Comparison of recurrence-free survival kidney allograft survival over 60 months post-transplant in the three subgroups of patients with C3 glomerulonephritis (C3GN, *n* = 12), dense deposit disease (DDD, *n* = 5), or immune complex membranoproliferative glomerulonephritis (IC-MPGN, *n* = 3)

**Fig. 2:**
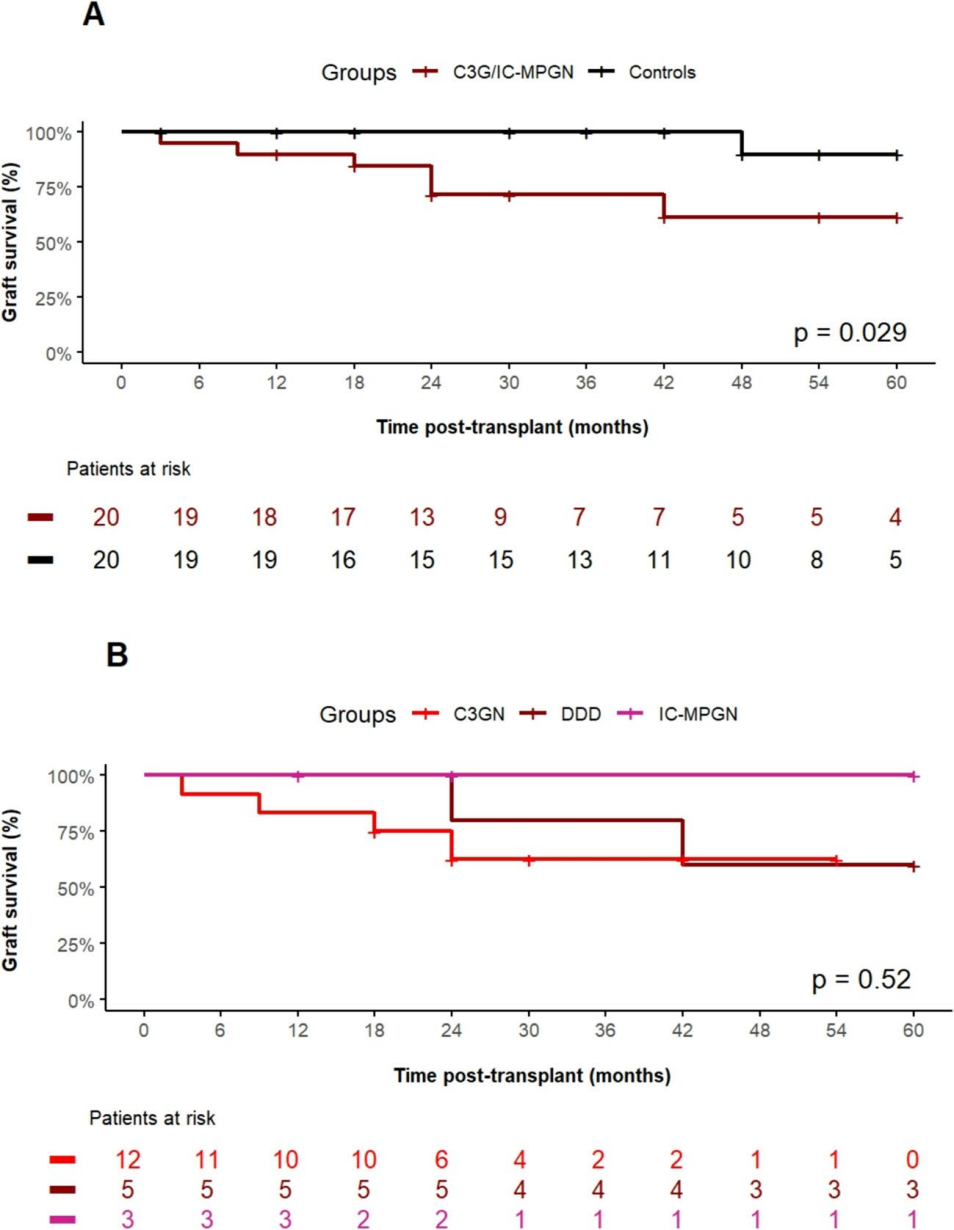
**A** Kidney allograft survival over 60 months post-transplant in patients with C3 glomerulopathy (*n* = 17) or immune complex membranoproliferative glomerulonephritis (*n* = 3) compared to matched controls (*n* = 20). There was a statistically significant difference (*P* = 0.029, log-rank test). **B** Comparison of kidney allograft survival over 60 months post-transplant in the three subgroups of patients with C3 glomerulonephritis (C3GN, *n* = 12), dense deposit disease (DDD, *n* = 5), or immune complex membranoproliferative glomerulonephritis (IC-MPGN, *n* = 3). There was no statistically significant difference (*P* = 0.52, log-rank test)

**Fig. 3:**
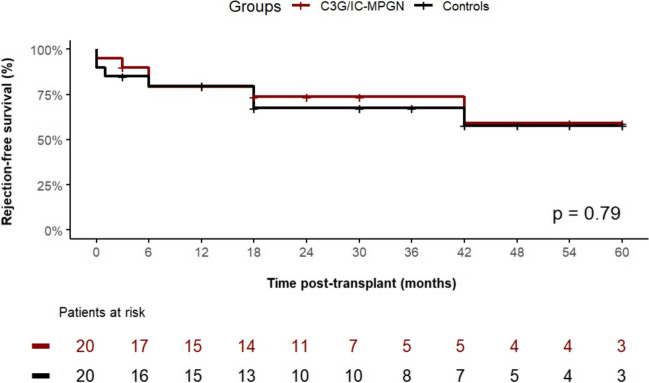
Acute rejection-free survival over 60 months post-transplant in patients with C3 glomerulopathy (*n* = 17) or immune complex membranoproliferative glomerulonephritis (*n* = 3). There was no statistically significant difference (*P* = 0.79, log-rank test)

Regarding post-transplant outcome data, the rate of delayed graft function, defined as the need for dialysis therapy within the first week post-transplant and the duration of initial hospitalization after transplant surgery, was comparable between the groups (Table 3). Figure 1 A shows the rate of recurrence of C3G or IC-MPGN post-transplant. Eleven of 20 children (55%) experienced recurrence within 60 months post-transplant. The respective recurrence rate did not significantly differ among C3GN (*n* = 8 of 12), DDD (*n* = 2 of 5), and IC-MPGN (*n* = 1 of 3) patients (Fig. 1 B). Figure 2 A shows the graft survival over 5 years post-transplant in patients with C3G or IC-MPGN compared to the matched control group. Patients with C3G or IC-MPGN had a 5-year graft survival of only 61.4%, which was significantly (*P* = 0.029) lower than the 5-year graft survival of 90% in matched controls. Graft survival was similarly reduced in patients with C3GN, DDD, and IC-MPGN (Fig. 2 B). Six patients with C3G or IC-MPGN lost their graft within the observation period of 5 years; in five of these 6 patients, recurrence of C3G was the primary cause of graft loss. No patient with IC-MPGN suffered a graft loss. In both groups, no deaths were reported. Figure 3 shows the number of biopsy-proven acute rejection (BPAR) episodes in patients with C3G or IC-MPGN compared to matched controls. Both the 1-year (20%) and the 5-year rate of BPAR (42% in the C3G and IC-MPGN cohort, 44% in the control cohort) were comparable between patients and controls. In those patients who did not lose their graft during the observation time of 5 years, transplant function as assessed by eGFR at months 1, 3, and 12 and at years 3 and 5 post-transplant was comparable between C3G or IC-MPGN patients and matched controls (Table 3).

**Table 3:** Comparison of outcome parameters of patients with C3G or IC-MPGN and matched controls

Outcome and endpoints	C3G/IC-MPGN cohort, *N* = 20	Matched controls, *N* = 20	*P v*alue
Delayed graft function, *n* (%)	3 (15.0)	1 (5.0)	0.29^b^
Time to discharge after KTx (days), mean ± SD	20.8 ± 10.2	19.7 ± 10.1	0.75^a^
Graft loss, event-free survival at month 60 post-transplant (estimate ± SE) [95% confidence interval]	0.61 ± 0.13 [0.40–0.94]	0.90 ± 0.09 [0.73–1.00]	0.029^c^
Death, *n* (%)	0 (0.0)	0 (0.0)	-
Biopsy-proven acute rejection episodes, event-free survival at month 60 post-transplant (estimate ± SE)^1^ [95% confidence interval]	0.59 ± 0.15 [0.35–0.98]	0.58 ± 0.13 [0.37–0.89]	0.79^c^
Kidney transplant function as assessed by eGFR (ml/min per 1.73 m^2^), mean ± SD			
Month 1	69.0 ± 29.8, *N* = 20	75.5 ± 25.7, *N* = 20	0.47^a^
Month 3	70.3 ± 25.6, *N* = 20	69.8 ± 16.8, *N* = 20	0.94^a^
Month 12	67.6 ± 18.1, *N* = 18	76.3 ± 30.9, *N* = 19	0.31^a^
Month 36	62.0 ± 26.2, *N* = 7	62.1 ± 23.8, *N* = 13	0.99^a^
Month 60	66.0 ± 18.6, *N* = 4	79.8 ± 38.5, *N* = 5	0.51^a^

^1^Biopsy-proven rejection episodes: one event is defined as time to first biopsy-proven rejection episode per patient

Abbreviations: *eGFR*, estimated glomerular filtration rate. Statistical tests: ^a^parametric *t*-test; ^b^chi-square test; ^c^log-rank test

Table 4 shows the individual follow-up data post-transplant. In case of recurrence, patients with C3G or IC-MPGN were treated with more intense maintenance immunosuppressive therapy. Twelve of 20 (60%) patients received eculizumab post-transplant, either as prophylaxis or as newly initiated rescue therapy in case of recurrence. Eight patients received eculizumab prophylactically; in 5 of these 8 patients (62.5%), C3G recurred despite this treatment. One of these 5 patients was also treated with immunoadsorption prior to recurrence but not at recurrence and not in the further follow-up period. This patient did not lose his graft due to recurrence. Four patients received eculizumab for the treatment of recurrence; 1 of these 4 patients (25%) lost the kidney transplant due to recurrence despite that treatment. One of these 4 patients who were treated with eculizumab following recurrence had also received plasma exchange at recurrence but not in the further follow-up period; this patient did not lose his graft due to recurrence. No patient received rituximab post-transplant.

**Table 4:** Characteristics of patients with C3G or IC-MPGN at time of kidney transplantation and thereafter

ID	Disease subtype	Sex	Age at KTx	Donor source	Recurrence post-transplant	Indication for KTx biopsy	Lowest reported serum C3 level^1^	Rituximab	PE/IA	Complement-targeted therapy	Graft loss	Loss to follow-up
1	C3GN	f	15.3	DD	Within 15 days	Proteinuria and hematuria	0.68 mg/dl at time of recurrence	No	PE at recurrence	At recurrence	No	Month 24
2	C3GN	m	9.8	DD	Month 3	Graft dysfunction	0.58 mg/dl 2 months before recurrence	No	No	Before recurrence	Month 9 (recurrence)	Month 12
3	C3GN	m	16.3	LD	No	NA	0.47 mg/dl	No	No	Before KTx	No	Month 36
4	C3GN	m	17.1	DD	Month 3	ND	ND	No	ND	ND	Month 3 (recurrence)	Month 6
5	C3GN	m	18.2	DD	Month 12	Protocol biopsy	0.47 mg/dl 1 month before recurrence	No	No	Before recurrence	No	Month 36
6	C3GN	m	13.3	DD	No	NA	1.31 mg/dl	No	No	Month 6 till month 18 post-KTx	No	Month 24
7	C3GN	f	15.9	LD	Month 1	Nephritic/nephrotic syndrome	0.19 mg/dl	No	No	At recurrence	Month 18 (recurrence)	Month 24
8	C3GN	f	15.6	LD	No	NA	1.0 mg/dl	No	No	No	No	Month 24
9	C3GN	f	16.8	DD	Month 12	ND	0.19 mg/dl 3 months before recurrence	No	No	Before recurrence	No	Month 30
10	C3GN	f	6.1	DD	Month 3	Protocol biopsy	0.35 mg/dl	No	IA before recurrence	Before recurrence	No	Month 60
11	C3GN	f	16.5	LD	No	NA	0.25 mg/dl	No	No	Before KTx and till month 6 post-KTx	No	Month 48
12	C3GN	m	15.3	LD	Within 15 days	Nephrotic syndrome	0.72 mg/dl	No	No	Before recurrence and KTx	Month 24 (recurrence)	Month 30
13	DDD	f	14.7	DD	No	ND	0.42 mg/dl	No	No	No	Month 42	Month 48
14	DDD	m	5.6	DD	Month 1	Proteinuria and haematuria	0.75 mg/dl	No	No	At recurrence	No	No
15	DDD	f	8.8	DD	No	NA	0.41 mg/dl	No	No	Before KTx and till year 6 post-KTx	No	No
16	DDD	f	11.6	DD	No	NA	2.01 mg/dl	No	No	No	No	Month 78
17	DDD	f	13.1	LD	Month 12	Proteinuria and haematuria	0,24 mg/dl	No	No	No	Month 24 (recurrence)	Month 30
18	IC-MPGN	m	10.2	DD	No	NA	0.76 mg/dl	No	ND	No	No	No
19	IC-MPGN	m	18.4	DD	No	NA	0.78 mg/dl	No	ND	No	No	Month 30
20	IC-MPGN	f	17.2	DD	Month 1	ND	0.59 mg/dl 1 month before recurrence	No	No	At recurrence	No	Month 18

^1^Levels in closest proximity to the event in case of recurrence are given. In patients without recurrence, the lowest C3 level during the observation period is given

*C3G*, C3 glomerulopathy; *C3GN*, C3 glomerulonephritis; *LD*, living donor; *DD*, deceased donor; *f*, female; *m*, male; *IA*, immunoadsorption; *PE*, plasma exchange; *KTx*, kidney transplantation; *NA*, not applicable; *ND*, not determined

## Discussion

We report here the largest series to date of pediatric patients with C3G or IC-MPGN who had received a KTx, because their native kidney function had failed. We observed a high risk of recurrence (55%) within the first 5 years after KTx, which was comparable to the risk of recurrence observed in previously published adult cohorts [9]. A recent study from the GLOSEN group reporting outcomes in adult C3G and IC-MPGN patients following KTx showed that 81 patients (37%) reached kidney failure at a median follow-up of 79 months. Disease recurrence was a key driver of this, occurring in 62% of patients with C3G and 15% of patients with idiopathic IC-MPGN [21]. Zand et al. investigated recurrence of C3GN after KTx specifically in adolescents and adult patients with protocol biopsies performed at month 4 and years 1, 2, 3, and 10 after transplantation. The disease recurred in 66.7% of all cases, resulting in a graft-loss rate of 50% [22]. An earlier pediatric study on MPGN in general found an increased rate of graft loss (32.4%) in children within 5 years after KTx. Recurrence was not specifically investigated [23]. In our cohort, the respective recurrence rate did not significantly differ among C3GN, DDD, and IC-MPGN patients, but the numbers were rather small for a valid statistical comparison. A variety of therapies were used either for prophylaxis or treatment of recurrent C3G or IC-MPGN. Eight patients received eculizumab prophylactically; however, in 5 of these 8 patients (62.5%), C3G recurred nevertheless. These data confirm the previous observation that inhibition of the terminal complement pathway with a C5-targeting antibody is not sufficient for the treatment of C3G or IC-MPGN either in native disease [24] or in case of recurrence in the transplanted kidney [9], and underscores the urgent medical need for more effective therapies for these diseases.

One unique feature of the present study is the comparison of post-transplant outcome data of C3G or IC-MPGN patients with a well-matched control group of patients with non-glomerular (and hence non-recurring) primary kidney diseases. We observed that patients with C3G or IC-MPGN had a significantly lower 5-year graft survival than matched controls with other primary kidney diseases, and this lower graft survival was mainly due to recurrence and not due to rejection, because the rate of BPAR was comparable between C3G or IC-MPGN patients and matched controls. Hence, children and adolescents with C3G or IC-MPGN have a significantly shorter kidney transplant survival rate, due to the recurrence of their respective underlying disease, than patients of the same age with other primary kidney diseases, and therefore cannot benefit equally from KTx. This disadvantage is even more severe in children and adolescents than in adults, because pediatric patients are particularly dependent on the longest possible kidney transplant survival rate due to their significantly higher life expectancy.

There is no targeted therapy addressing the underlying pathophysiology of either C3G or IC-MPGN. Both diseases have recently been discussed in a KDIGO Controversies Conference [25]. Due to the current lack of randomized controlled trials for the treatment of C3G and IC-MPGN, KDIGO has published practice points for treatment, which are based on expert opinion, a limited number of retrospective studies, and extrapolation from other proliferative glomerulonephritides. These practice points focus on the management of primary disease but the level of evidence supporting the use of recommended therapies, including RAAS inhibition, corticosteroids, and MMF/MPS, is low [24]. Our data show that C3G in pediatric KTx recipients did recur despite immunosuppressive treatment with MMF and/or corticosteroids. Furthermore, KDIGO made no specific recommendations regarding the management of recurrent disease [24]. There is therefore great need for a specific therapy, which directly targets the underlying disease mechanism. After the recent successful approval of the C3 inhibitor pegcetacoplan for the treatment of paroxysmal nocturnal hemoglobinuria [26], iptacopan, a novel, low molecular weight, orally active, reversible, and selective inhibitor of Factor B (FB) of the complement AP, is currently developed. Inhibition of Factor B selectively inhibits the catalytic activity of C3 and C5 convertases [27]. Further, the C5a-Receptor antagonist avacopan has been successfully applied to a child with C3GN who was enrolled in the ACCOLADE study (NCT03301467) [28]. Targeted inhibition of complement pathways is expected to be therapeutically beneficial across a range of diseases in which complement AP dysregulation plays an important role in pathogenesis, including C3G and IC-MPGN [29].

The strengths of our study are the multicenter design, the largest number of pediatric patients with C3G or IC-MPGN patients post-transplant analyzed to date, and the review of all kidney transplant biopsy reports by a single histopathologist. Our study has several limitations, the most important of which is the retrospective study design, which is common to registry analyses in general. Another limitation is the lack of centralized laboratory assessment, and the missing data on autoantibodies against complement factors and on mutations in genes encoding complement-regulating proteins in some patients. Also, quantitative data on proteinuria, which is an important risk factor for KTx outcome, were not reported by many centers. In addition, it was not possible to retrospectively assess patient adherence to immunosuppressive medications, which could affect outcome in both the control and C3G/IC-MPGN groups. However, registries reflecting the real-world scenario with heterogeneous immunosuppressive regimens and treatment protocols are potentially suitable to provide a broader view of outcomes and complications, especially in small cohorts such as pediatric kidney allograft recipients.

In conclusion, this multicenter registry study provides an estimate of the incidence of recurrence of C3G or IC-MPGN in the largest series to date of pediatric kidney transplant recipients. Our data show a high risk of recurrence (55%) within 5 years after KTx. This rate is comparable to the risk of recurrence reported in adults. Due to recurrence, pediatric patients with C3G or IC-MPGN had a significantly lower 5-year graft survival than matched controls with other primary kidney diseases. These data underscore the medical need for effective and specific medications both for the prophylaxis and treatment of these progressive and post-transplant recurring kidney diseases to improve the outcome of KTx in this vulnerable patient population.

## Supplementary Information

Below is the link to the electronic supplementary material.

Graphical abstract (PPTX 142 KB)

ESM 1 (DOCX 21.5 KB)

## Data Availability

The data that support the findings of this study are available from the corresponding author upon reasonable request.

## Abbreviations

AP: Alternative pathway
CAKUT: Congenital anomalies of the kidney and urinary tract
C3G: Complement C3 glomerulopathy
C3GN: C3 glomerulonephritis
CERTAIN: Cooperative European Paediatric Renal Transplant Initiative
DDD: Dense deposit disease
eGFR: Estimated glomerular filtration rate
HLA: Human leukocyte antigen
IC-MPGN: Immune complex–mediated glomerulonephritis
IQR: Interquartile range
KTx: Kidney transplantation
MMF: Mycophenolate mofetil
MPS: Mycophenolate sodium
RAAS: Renin–angiotensin–aldosterone system
SD: Standard deviation
