# Supplementary material for: Kidney transplantation in children and adolescents with C3 glomerulopathy or immune complex membranoproliferative glomerulonephritis: a real-world study within the CERTAIN research network
Source: Pediatr Nephrol. 2024 Aug 7;39(12):3569–80. doi: 10.1007/s00467-024-06476-5 (PMC11511764; doi:10.1007/s00467-024-06476-5)
Supplement: Supplementary file 1 — Graphical abstract (PPTX 142 KB) [file 467_2024_6476_MOESM1_ESM.pptx]

## Slide 1
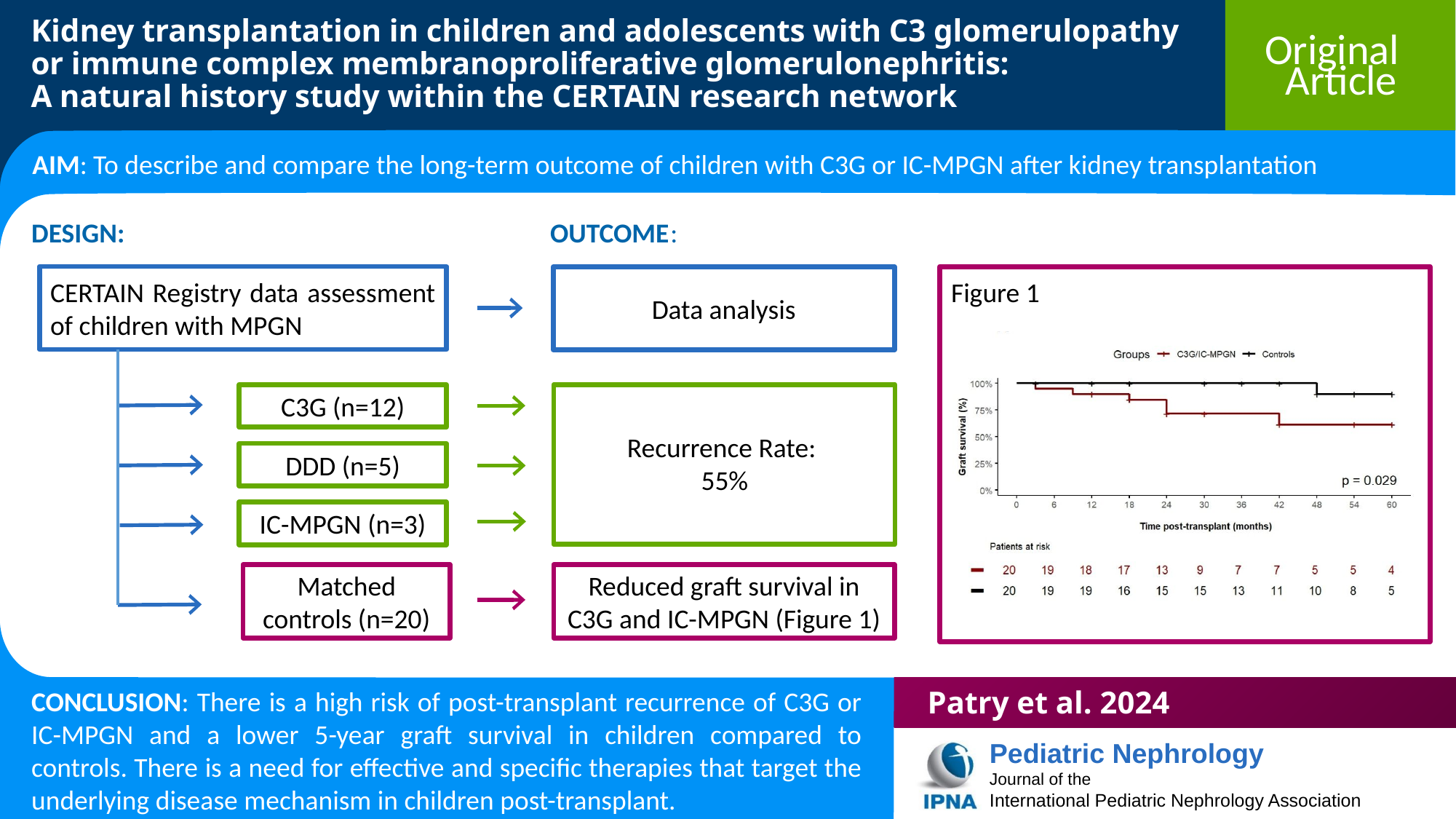

Kidney transplantation in children and adolescents with C3 glomerulopathy or immune complex membranoproliferative glomerulonephritis:
A natural history study within the CERTAIN research network
AIM: To describe and compare the long-term outcome of children with C3G or IC-MPGN after kidney transplantation
DESIGN: OUTCOME:
CERTAIN Registry data assessment of children with MPGN
Data analysis
Figure 1
C3G (n=12)
Recurrence Rate:
55%
DDD (n=5)
IC-MPGN (n=3)
Reduced graft survival in C3G and IC-MPGN (Figure 1)
Matched controls (n=20)
Patry et al. 2024
CONCLUSION: There is a high risk of post-transplant recurrence of C3G or IC-MPGN and a lower 5-year graft survival in children compared to controls. There is a need for effective and specific therapies that target the underlying disease mechanism in children post-transplant.
