# Supplementary material for: Kidney transplantation in children and adolescents with C3 glomerulopathy or immune complex membranoproliferative glomerulonephritis: a real-world study within the CERTAIN research network
Source: Pediatr Nephrol. 2024 Aug 7;39(12):3569–80. doi: 10.1007/s00467-024-06476-5 (PMC11511764; doi:10.1007/s00467-024-06476-5)
Supplement: Supplementary file 2 — (DOCX 21.5 KB) [file 467_2024_6476_MOESM2_ESM.docx]

**Supplemental Material:** Analysed parameters per patient according to data-entry in the CERTAIN-Registry.

**General data**

| Code of the reporting pediatric KTx center | **Visit date** |
| --- | --- |
| Lost to follow up (with date and reason)? | **Skipped visit?** |

**General data: Recipient and Donor**

| **Recipient** | **Donor** |
| --- | --- |
| Date of KTx | Age (years) |
| **Age (years)** | Sex |
| Sex | Donor type |
| Ethnic origin | Ethnic Origin |
| Number of previous KTx | HLA-A |
| **Primary kidney disease** | HLA-B |
| **Biopsy (if performed with result) of the native kidney?** | HLA-DRB1 |
| HLA-A |  |
| HLA-B |  |
| HLA-DRB1 |  |

**General data: Kidney Transplantation**

| Cold ischemia time (minutes) | Pretreatment of the recipient before KTx |
| --- | --- |
| Warm ischemia time (minutes) | HLA-A mismatches |
| Blood group compatibility | HLA-B mismatches |
| Biopsy before/during KTx | HLA-DRB1 mismatches |
| Date of discharge after KTx | Delayed Graft Function? |

**General data: At each visit**

| **Patient and Graft survival** | **Anthropometry** |
| --- | --- |
| Graft Loss | **Age (years)** |
| Death | Height (cm) |
| Cause of death | Weight (kg) |
| Graft Loss date | **BMI** |
| Death Date | **BSA** |
| Reason for graft Loss | **Height SDS** |
| Last biopsy of graft? | **BMI SDS** |
| Any rejection episode since the last visit? |  |
| Any graft biopsy since last visit? |  |

**Data assessment in case of any rejection episode**

| **Type of assessed data** | **Parameter name** |
| --- | --- |
| Signs and symptoms | Date of first suspected rejection |
|  | Suspicion raised by what signs and/or symptoms? (e.g. clinical, laboratory parameters) |
| Diagnostics | Biopsy performed? |
|  | Second biopsy performed? |
| Antirejection therapy | Steroids? |
|  | Antilymphocyte antibodies? |
|  | Change of maintenance immunosuppression (e.g. switch and/or dose increase?) |
|  | Intravenous immunoglobulins |
|  | Blood purification measures |
| Clinical course | Description of general outcome |

**Data assessment for Renal biopsies**

| Date of Biopsy | Lesion scores |
| --- | --- |
| Total number of glomeruli | g |
| Number of arteries | t |
| Adequacy of specimen (calculated) | i |
| Reason | v |
| Ultrasound | ci |
| Complication | ct |
| Serum creatinine at time of biopsy (mg/dl) | cg |
| Result of biopsy | mm |
| Pathology report available for upload | cv |
| **Banff version year** | ah |
| Antibody-mediated changes | ptc |
| T-cell mediated rejection | ti |
| Interstitial fibrosis and tubular atrophy | pvl |
| BK nephropathy | iIfta |
| Other findings | tIfta |
| C4d technique | Additional diagnostic parameters |
| C4d positivity |  |
| Scoring of C4d staining |  |

**Laboratory overview at each visit**

| **Type of assessed data** | **Parameter** |
| --- | --- |
| Graft Function | Serum creatinine (mg/dl) |
|  | GFR (ml/min/1.73m^2^) |
|  | Serum urea (mg/dl) |
|  | Serum Cystatin C (mg/l) |
| Urine chemistry | Urinary albumin/creatinine ratio (g/mol) |
|  | Urinary protein/creatinine ratio (g/mol) |

**Immunosuppressive medication and Plasma-therapy assessed at each visit**

| ATG | Cyclosporine microemulsion | Enteric-coated mycophenolate sodium |
| --- | --- | --- |
| ALG | Cyclosporine | Azathioprine |
| OKT3 | Tacrolimus | Prednisone |
| Basiliximab | Tacrolimus delayed release | Prednisolone |
| Belatacept | Sirolimus | Methylprednisolone |
| Daclizumab | Everolimus | Deflazacort |
| Rituximab | Mycophenolate mofetil | **Rituximab** |
| Immunoadsorption | Plasmapheresis |  |

**C3G-Study specific data set**

| **Pre-KTx-Data** | **Post-KTx-Data (each visit)** |
| --- | --- |
| Disease subtype (C3GN, DDD, IC-MPGN) | Complement C3 levels (mg/dl) |
| Have auto-antibodies been measured (specify if yes) | Treatment with complement inhibitor? |
| C3NeF | Eculizumab |
| C4NeF | Ravulizumab |
| C5NeF | Recurrence of primary kidney disease post KTx? |
| AntiFB Ab | Clinical presentation of recurrence (description) |
| AntiC3b Ab | Have auto-antibodies been measured? |
| AntiFH Ab | C3NeF |
| Other auto-antibodies (specify)? | C4NeF |
| Locus of mutation/genetic variant | C5NeF |
| Complement-Inhibitor-Treatment prior to KTx? | AntiFB Ab |
| Eculizumab | AntiC3b Ab |
| Ravulizumab | AntiFH Ab |
| Other treatment (specify)? | Other auto-antibodies (specify)? |
| Upload of pathology reports of native kidney | Upload of pathology reports of biopsies post KTx |
